# Identification of hepadnavirus in the sera of cats

**DOI:** 10.1038/s41598-019-47175-8

**Published:** 2019-07-23

**Authors:** Gianvito Lanave, Paolo Capozza, Georgia Diakoudi, Cristiana Catella, Leonardo Catucci, Paola Ghergo, Fabio Stasi, Vanessa Barrs, Julia Beatty, Nicola Decaro, Canio Buonavoglia, Vito Martella, Michele Camero

**Affiliations:** 1 Department of Veterinary Medicine, University of Bari, Valenzano, Italy; 2 ACV Triggiano srl, Triggiano, Italy; 3 Biotechlab, Brindisi, Italy; 4 University of Sydney, Sydney, Australia

**Keywords:** Viral epidemiology, Viral infection, Hepatitis B virus

## Abstract

Hepadnaviruses infect several animal species. The prototype species, human hepatitis B virus (HBV), increases the risk of liver diseases and may cause cirrhosis and hepatocellular carcinoma. Recently a novel hepadnavirus, similar to HBV, has been identified through transcriptomics studies in a domestic cat with large cell lymphoma in Australia. Herewith, a collection of 390 feline serum samples was screened for hepadnavirus. Overall, the virus was identified in 10.8% of the sera with a significantly higher prevalence (17.8%) in the sera of animals with a clinical suspect of infectious disease. Upon genome sequencing, the virus was closely related (97.0% nt identity) to the prototype Australian feline virus Sydney 2016. The mean and median values of hepadnavirus in the feline sera were 1.3 × 10^6^ and 2.1 × 10^4^ genome copies per mL (range 3.3 × 10^0^–2.5 × 10^7^ genome copies per mL). For a subset of hepadnavirus-positive samples, information on the hemato-chemical parameters was available and in 10/20 animals a profile suggestive of liver damage was present. Also, in 7/10 animals with suspected hepatic disease, virus load was >10^4^ genome copies per mL, i.e. above the threshold considered at risk of active hepatitis and liver damage for HBV.

## Introduction

Viruses of the genus *Orthohepadnavirus*, family *Hepadnaviridae*, are partially double-stranded DNA viruses that infect a variety of mammals. Chronic infections in humans of the prototype species, hepatitis B virus (HBV), increase the risk of liver diseases including cirrhosis and hepatocellular carcinoma^1^.

Hepadnaviruses have been identified in several animal species including primates, bats, rodents, birds and fish^2^. Recently a novel member of the family *Hepadnaviridae*, similar to HBV, has been identified through transcriptomics studies in a domestic cat with large cell lymphoma^3^. Preliminary epidemiological data collected by Australian researchers suggest that the hepadnavirus of domestic cat (DCH) is common in cats infected with feline immunodeficiency virus (FIV)^3^. In order to gather additional information on DCH, we analyzed sera collected from household cats with different age (0–15 years) and clinical histories, obtained from veterinary diagnostic laboratories.

## Results and Discussion

**Figure 1:**
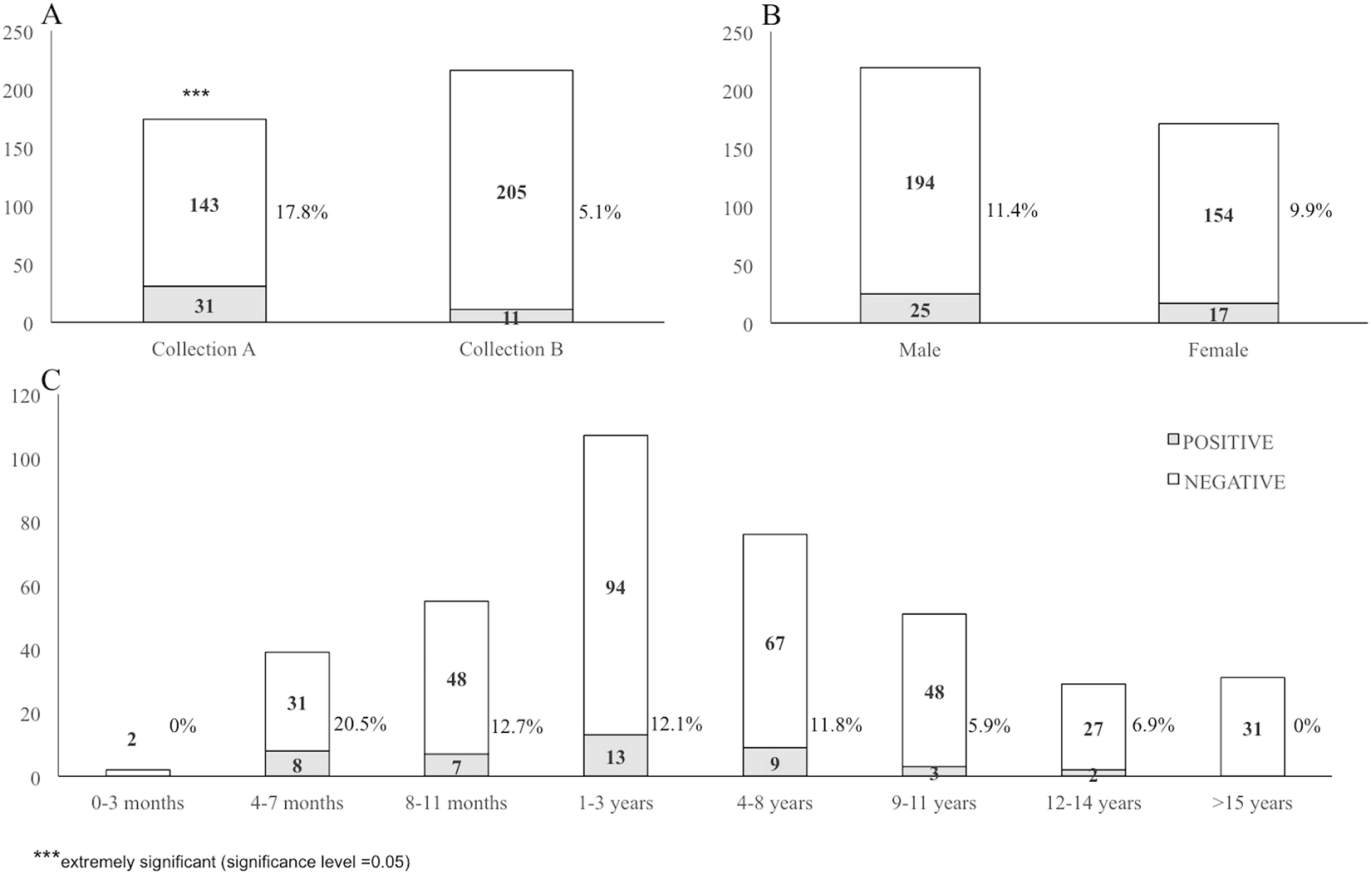
Results of the screening for DCH in the feline sera. The prevalence of DCH was evaluated in sera collected for the diagnosis of infectious diseases (collection **A**) and sera submitted to the laboratory for pre-surgical evaluation or for suspected metabolic or neoplastic disease (collection **B**) and used for comparison (panel A). The prevalence of DCH was also evaluated in relation to sex (panel B) and age (panel C) of the animals.

Overall, we detected hepadnavirus DNA in 42/390 (10.8%) sera. DCH DNA was detected in 31 (17.8%) out of 174 sera collected with a request for diagnosis of infectious diseases (collection A) and in 11 (5.1%) out of 216 sera submitted to the laboratory for pre-surgical evaluation or for suspected metabolic or neoplastic disease (collection B) that were used to generate a baseline. The difference for DCH prevalence was statistically extremely significant (p-value = 0.00006, OR = 4.04005, CI95% = [1.96601; 8.30211]) when compared animals from collection A and collection B (Fig. 1A). Moreover, there was no significant difference in terms of prevalence (p = 0.64125) between male (11.4%, 25/219) and female (9.9%, 17/171) individuals (Fig. 1B). We also analysed the distribution of hepadnavirus across various age groups (Fig. 1C). The prevalence of DCH was higher in cats aged 4 to 7 months (20.5%, 8/39) although without statistically significant difference (p = 0.08061) compared to other age groups.

**Figure 2:**
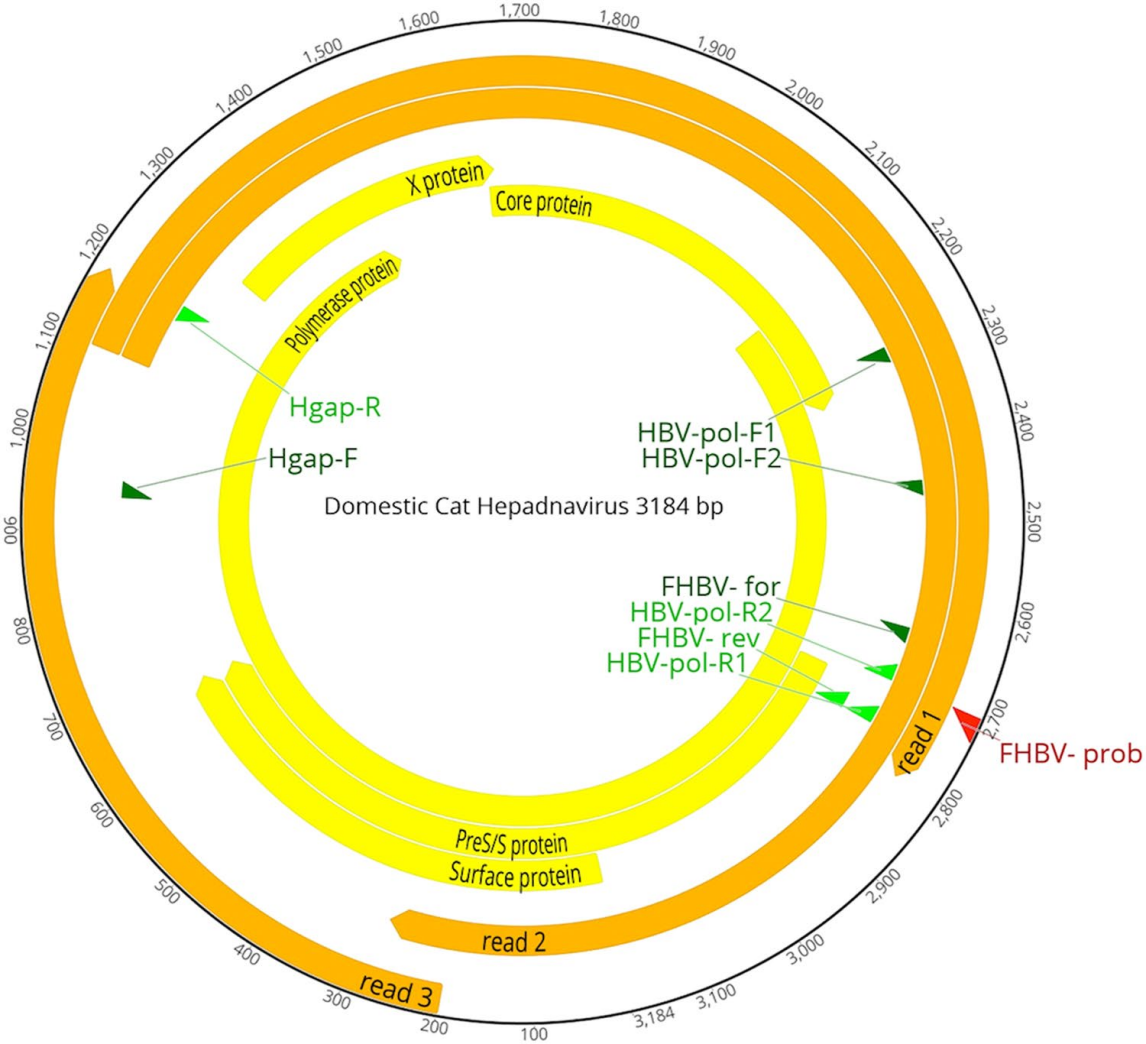
Genome organization of the DCH. The complete genome consists of 3184 bp. The outermost circles represent the read mapping coverage (orange) of the genome. In the innermost circles the proteins encoded by the polymerase, surface, core and X ORFs are labelled (yellow), as are the positions of the primers (green) and probe (red) used in this study (Table 2) are indicated.

**Figure 3:**
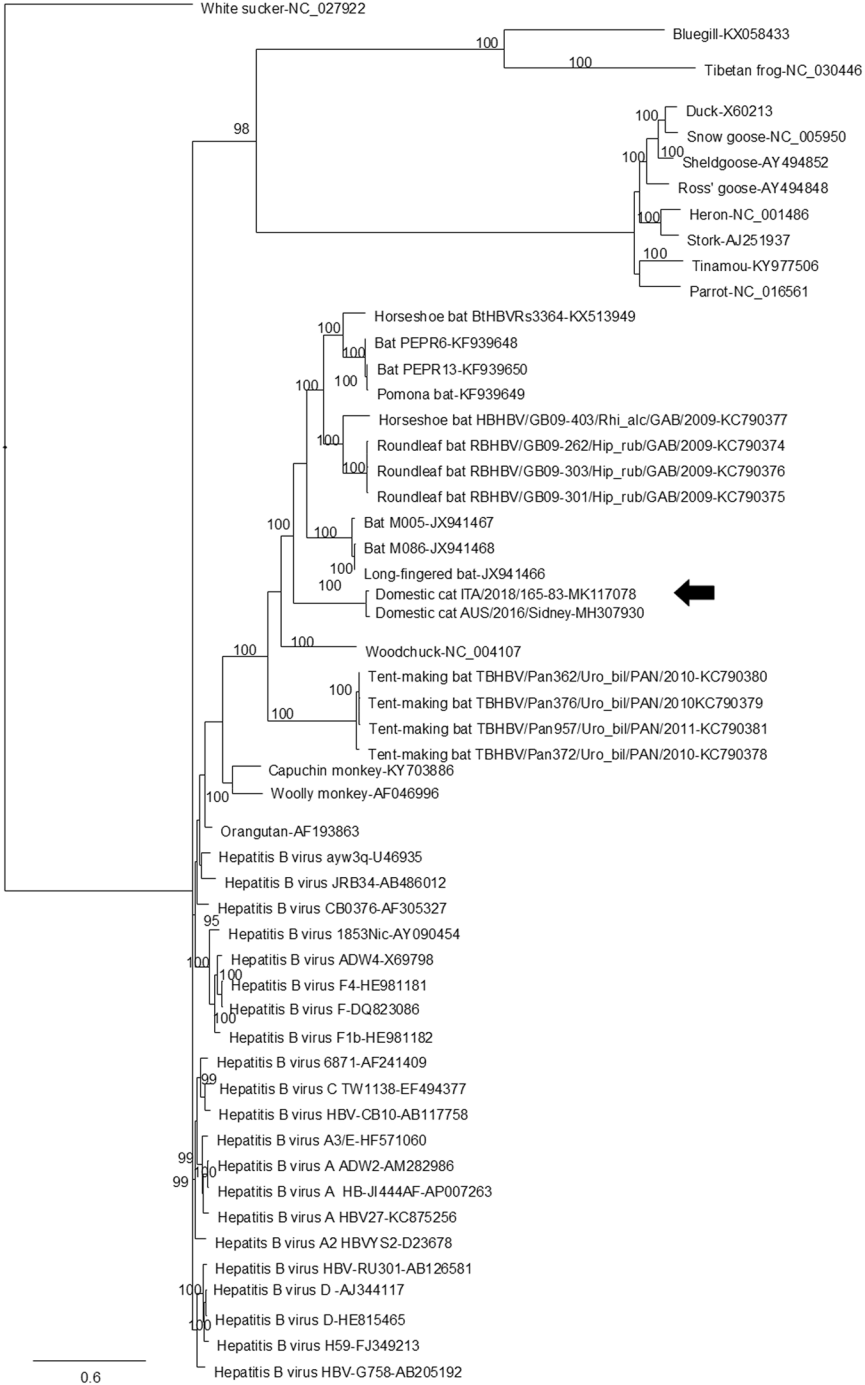
Bayesian phylogenetic tree based on complete genomic sequences of hepadnaviruses retrieved from the Genbank database. Posterior probability values >95% are shown on the nodes of the tree. Italian feline strain ITA/2018/165-83 (GenBank accession nr. MK117078) is indicated by black arrow. White Sucker hepadnavirus (NC_027922) was used as outgroup. The scale bar indicates the number of nucleotide substitutions per site.

We reconstructed the complete genome of the DCH strain ITA/2018/165-83, of approximately 3.2 kb (Fig. 2), which had 97.0% nucleotide sequence identity to the Australian reference strain AUS/2016/Sydney across the entire genome. Some differences were observed in the tree topologies between our analysis and previous studies^2^, likely accounted for by the alignment complexity. In the consensus phylogenetic tree (Fig. 3), a large group included bat and rodent viruses. The two feline hepadnaviruses segregated together in a defined branch within this large group. All the primate viruses were basal to this bat/rodent/feline group. Also, there were two additional well-defined groups, encompassing avian and amphibian viruses, respectively.

The findings of this study confirm that the novel hepadnavirus is a common component of the feline virome. The pathogenic role of this hepadnavirus, if any, is not yet known, but thus far, all hepadnaviruses have been reported to replicate preferentially in hepatocytes^1,4^. In this study we observed a marked and significantly higher prevalence (17.8%, 31/174) in the cohort of cats with suspected infectious diseases (collection A) with respect to a group of animals (collection B) used as baseline. Almost half of the sera positive for DCH (14/31, 45.2%) of this cohort were collected from cats with retroviral infection (FIV and/or feline leukemia virus, FeLV). In turn, the overall prevalence of retroviral infection in collection A was 24.1% (42/174), with 33.3% (14/42) being co-infections with DCH with a statistically significant (p = 0.0048) correlation between DCH and retrovirus positive cats. These findings echo what has been documented for HBV, which is more frequently observed in immunocompromised individuals^5^. Reactivation of HBV is common in patients with immunosuppression^5^. Even more interestingly, hepatopathy has been described in FeLV-infected cats, with icterus and various inflammatory and degenerative liver diseases^6^. Feline retroviruses have been demonstrated to impair severely the immune system in infected cats and diseases associated with immune-suppression account for a large portion of the morbidity and mortality observed in FeLV-infected cats^7–9^.

Transmission of HBV in humans occurs through blood and other body fluids and contagion can also occur during sexual contact and by maternal/fetal route^1,4^. Transmission of FIV/FeLV in cats occurs with similar modalities, as the virus is present in blood and body fluids^9^. Similar modalities of transmission might also be hypothesized for DCH, since we found viremia in 10.8% of the cats. Importantly, the presence of DCH in the sera may pose unexpected risks in transfusion medicine. DCH, along with feline retroviruses, *Bartonella* spp. and feline hemoplasma, should be considered in the screening of donor subjects^10^.

In cats there are not known viral agents strictly associated with hepatic disease, unlike what observed in human medicine where there are five major types of viral hepatitis (A to E)^11^. For diagnosis of HBV infection and for predicting the stage of infection in human patients, antigens, antibodies and viral genome are profiled/quantified and this information is coupled with haematological and blood chemistry tests. In the case of DCH, we can only use the molecular diagnostics and hematological and blood chemistry data, since there are no immunological reagents available for the diagnostics. Out of 42 DCH-infected cats, we could retrieve information on hematologic and serum biochemical parameters for 20 animals (Table 1). In 10 of these, increased levels of markers indicative of structural or functional liver damage (i.e. AST, ALT, ALP, GGT and total bilirubin) were present. HBV load is considered relevant in infected human patients and varies markedly across the phases of HBV infection^1^. The lower threshold for risk of active hepatitis and liver damage is 10^4^ viral genome equivalents of HBV per mL, equivalent to about 2000 IU (international unit)/mL^1^. Usually, high HBV DNA load in blood is found in acute infection, or in active chronic stages of disease, but the virus can reactivate after long periods of apparent remission, chiefly in immunosuppressed patients^1^. The mean and median values of DCH viremia in feline sera were 1.3 × 10^6^ and 2.1 × 10^4^ DNA copies per mL (range 3.3 × 10^0^–2.5 × 10^7^ DNA copies per mL). In 7 out of 10 animals with suspected hepatic disease, DCH load was >10^4^ genome copies per mL. Although this parallelism between HBV and DCH is intriguing, whether a correlation also exists between DCH replication and liver damage should be assessed in structured, larger observational studies. For instance, we also found the virus in cats with unaltered hepatic markers. This condition occurs in human patients with chronic HBV infection during the inactive “immune‐control” phase, defined as the presence of HBV antigens in serum without ALT elevation and with low viremic HBV‐DNA levels^12^. However, only in 3/10 cats with non-altered hepatic markers the virus titer was lower than 10^4^. The actual patho-biology of DCH in cats should be determined in order to understand better the patterns of DCH infection.

**Table 1:** List of DCH-positive animals with the corresponding hemato-chemicals parameters.

Animal	Gender	Age Months	RBC	WBC	PLT	CPK	AST	ALT	ALP	GGT	Total Bilirubin	FIV and/or FeLV	N° viral DNA copies/ml
			6.0–9.5 millions/μL	5.5–12 ×1000/μL	130–400 1000/μL	100–350 UI/L	10–55 UI/L	10–75 UI/L	20–100 UI/L	0–2 UI/L	0.00–0.20 mg/dL		
418/17-8*	M	24				1194		77				−	8,14*10^5^
914/17 - 13*	F	48	4.20	15.1		453						−	2,51*10^3^
914/17 – 43*	F	96	4.36					**140**	**10**			−	2,5*10^7^
165/18 – 101*	M	108		36.2	509		**204**	**245**	**185**	2.4	**18**.**22**	−	117*10^4^
510/18 – 6*	M	36				607	**203**	**120**				−	1.43*10^5^
**377/17 - 18**	M	24	2.95		33		**177**		**151**			**+**	7.85*10^1^
165/18 - 83	M	36	3.91	3.0	38	615	59				7.89	+	2.74*10^6^
169/18 - 4	F	24	4.43	13.9		410			12	2.2		+	2.25*10^4^
255/18 - 20	M	7	4.27	20.7		363			10			+	2.0*10^4^
**296/17 - 20**	F	156		17.8		1566	**205**	**152**	**110**		**1**.**27**	−	1.46*10^5^
**342/17 - 11**	M	6	9.73	15.9		432	**284**	**261**			**0**.**24**	−	1.80*10^4^
342/17 - 21	M	60				789			120			−	9.77*10^4^
342/17 - 42	M	11		14.9		882			103			−	2.14*10^4^
**377/17 - 13**	F	7				775		**116**	**126**			−	1.96*10^4^
377/17 - 27	F	8		17.1	111							−	2.42*10^4^
**470/17 - 22**	F	7						**194**	**121**			−	2.51*10^2^
470/17 - 26	M	8	1.80	1.1		886			12		0.63	−	7.85*10^1^
513/17 - 10	F	12	4.37	56.7	490	1312	68		16		0.29	−	9.70*10^3^
**34/18 - 1**	F	132	10.35					**121**		2.5		−	2.24*10^4^
**255/18 - 21**	F	24					**71**	**114**				−	1.70*10^3^

Asterisks indicate group B animals. Animals with parameters indicative of functional and/or structural hepatic impairment are indicated in bold. Only parameters exceeding the normal ranges were reported in the table.

Several novel viruses have been discovered in cats in recent years^13,14^. Optimizing the diagnostic algorithms and gathering epidemiological data will help assessing the possible pathogenic role of these viruses in cats and eventually conceive strategies to protect their health.

## Methods

A total of 390 sera were collected from two different veterinary clinic laboratories located in Apulia region, Southern Italy and tested upon request of the veterinarian practitioners after anamnesis, medical history and clinical examination. One-hundred seventy-four sera (collection A) had been collected for diagnosis of infectious diseases (FIV, FeLV, feline coronavirus (FCoV), toxoplasmosis, hemoplasmosis, bacterial and fungal infections). A total of 147/174 (84.5%) sera were submitted with a suspect/request of diagnosis for FIV/FeLV, with 42 sera being positive for retrovirus. Fisher’s exact test was performed to the collection A to evaluate the correlation between DCH positive cats and retrovirus positive cats. The significance level of the test was set at 0.05. Of the other 27 sera (15.5%), 14 were sent for a suspect/request of diagnosis for coronavirus (with 7/14 being positive), 5 for toxoplasmosis (with 2/5 being positive), 2 for giardia (with 1/2 being positive). Five sera were collected from animals with suspected bacterial/fungal infections and 1 serum (negative) was from a cat with suspected hemoplasmosis. A total of 216 sera (collection B) submitted to the laboratory for pre-surgical evaluation (n = 85) or for suspected metabolic (n = 127) or neoplastic (n = 4) disease was used for comparison to generate a baseline. Information on the sera analyzed in the study is included in Fig. 1. The study was approved by the Ethics Committee of the Department of Veterinary Medicine, University of Bari (authorization 23/2018). All experiments were performed in accordance with relevant guidelines and regulations.

Total DNA was extracted from collected sera by using QIAamp cador Pathogen Mini Kit (QIAGEN, Hilden, Germany), according to the manufacturer’s instructions. We performed sample screening using a PCR with consensus pan-hepadnavirus primers^2^ and a PCR with primers specific for DCH^3^. Also, we screened sera using a quantitative PCR (qPCR) designed based on the sequence of the Australian reference strain AUS/2016/Sydney (GenBank accession nr. MH307930) (Table 2). For qPCR, we calculated DCH DNA copy numbers on the basis of standard curves generated by 10-fold dilutions of a plasmid standard TOPO XL PCR containing a 1.4 kb long fragment of the polymerase region of the Australian reference strain AUS/2016/Sydney (IQ Supermix; Bio-Rad Laboratories SRL, Segrate, Italy). We added 10 μL of sample DNA or plasmid standard to the 15-μL reaction master mix (IQ Supermix; Bio-Rad Laboratories SRL, Segrate, Italy) containing 0.6 μmol/L of each primer and 0.1 μmol/L of probe. Thermal cycling consisted of activation of iTaq DNA polymerase at 95 °C for 3 min and 42 cycles of denaturation at 95 °C for 10 s and annealing-extension at 60 °C for 30 s. We evaluated the specificity of the assay using a panel of feline DNA viruses (parvovirus, herpesvirus and poxvirus). The qPCR assay was able to detect as few as 10^1^ DNA copies per mL of standard DNA and 3.3 × 10^0^ DNA copies per mL of DNA template extracted from clinical samples. DCH quantification displayed acceptable levels of repeatability over a range of target DNA concentrations, when calculating the intra- and inter-assay coefficients of variation within and between runs, respectively^15,16^.

**Table 2:** Primer/probes used in this study.

Assay	Primer	Sequence 5′ – 3′	Amplicon size	Tm (°C)	Reference
PCR with consensus pan-hepadnavirus primers	HBV-pol-F1	TAGACTSGTGGTGGACTTCTC	593	45	Wang *et al*.^2^
	HBV-pol-R1	CATATAASTRAAAGCCAYACAG			
	HBV-pol-F2	TGCCATCTTCTTGTTGGTTC	258	45	
	HBV-pol-R2	AGTRAAYTGAGCCAGGAGAAAC			
PCR with specific primers for DCH	Hgap-F	GTGCTCTGATAACCGTATGCTC	230	55	Aghazadeh *et al*.^3^
	Hgap-R	CTAGAATGGCTACATGGGGTTAG			
Quantitative PCR (qPCR)	FHBV- for	CGTCATCATGGGTTTAGGAA	105	50	This study
	FHBV- rev	TCCATATAAGCAAACACCATACAAT			
	FHBV- prob	[FAM]TCCTCCTAACCATTGAAGCCAGACTACT [BHQ]			

We carried out inferential statistical analyses using the Chi-Squared test with Yates’ Correction, the evaluation of the odds ratio (OR) and 95% confidence interval (CI95%) with the online software MedCalc easy-to-use Statistical software (https://www.medcalc.org/calc/odds_ratio.php). The significance level of the test was set at 0.05.

Full genome sequences of hepadnaviruses were retrieved from the GenBank database and aligned using Geneious version 9.1.8 (Biomatters LTD, Auckland, New Zealand) and the MAFFT algorithm^17^. A set of genome sequences used in a previous study^2^ was integrated with additional genome sequences of hepadnaviruses of recent identification in mammalian, avian, amphibian species and in fish. The final dataset included 53 hepadnavirus genomes. Phylogenetic analysis was performed using JModel test (http://evomics.org/resources/software/molecular-evolution-software/modeltest/) to evaluate the correct best-fit model of evolution for the entire dataset. Bayesian analysis^18,19^ was therefore applied using four MCMC chains well-sampled and converging over one million generations (with the first 2000 trees discarded as “burn-in”) and supplying statistical support with subsampling over 1000 replicates. The identified program settings for all partitions, under the Akaike information criteria, included six-character states (general time-reversible model), a proportion of invariable sites and a gamma distribution of rate variation across sites (GTR + I + G). We also tried to perform phylogenetic analyses using other evolutionary models (Maximum likelihood, Neighbor joining) to compare the topology of phylogenetic trees. We could observe similar topologies with slight difference in bootstrap values at the nodes of the tree. Accordingly, we did prefer to retain the Bayesian tree. We deposited the nucleotide genome sequence of strain ITA/2018/165-83 (MK117078) in GenBank.

## Data Availability

All data generated or analyzed in this study are included in this published article.
